# Methodologic approach for the Biomarkers Reflecting Inflammation and Nutritional Determinants of Anemia (BRINDA) project

**DOI:** 10.3945/ajcn.116.142273

**Published:** 2017-06-14

**Authors:** Sorrel ML Namaste, Grant J Aaron, Ravi Varadhan, Janet M Peerson, Parminder S Suchdev

**Affiliations:** 1 Strengthening Partnerships, Results, and Innovations in Nutrition Globally, Arlington, VA;; 2 Helen Keller International, Washington, DC;; 3 Global Alliance for Improved Nutrition, Geneva, Switzerland;; 4 Department of Oncology, Johns Hopkins University, Baltimore, MD;; 5 Department of Nutrition, University of California, Davis, Davis, CA;; 6 Nutrition Branch, CDC, Atlanta, GA; and; 7 Department of Pediatrics and Global Health, Emory University, Atlanta, GA

**Keywords:** anemia, body iron, inflammation, iron, meta-analysis, vitamin A

## Abstract

**Background:** The Biomarkers Reflecting Inflammation and Nutritional Determinants of Anemia (BRINDA) project is a multiagency and multicountry collaboration that was formed to improve micronutrient assessment and to better characterize anemia.

**Objectives:** The aims of the project were to *1*) identify factors associated with inflammation, *2*) assess the relations between inflammation, malaria infection, and biomarkers of iron and vitamin A status and compare adjustment approaches, and *3*) assess risk factors for anemia in preschool children (PSC) and women of reproductive age (WRA).

**Design:** The BRINDA database inclusion criteria included surveys that *1*) were conducted after 2004, *2*) had target groups of PSC, WRA, or both, and *3*) used a similar laboratory methodology for the measurement of ≥1 biomarker of iron [ferritin or soluble transferrin receptor or vitamin A status (retinol-binding protein or retinol)] and ≥1 biomarker of inflammation (α-1-acid glycoprotein or C-reactive protein). Individual data sets were standardized and merged into a BRINDA database comprising 16 nationally and regionally representative surveys from 14 countries. Collectively, the database covered all 6 WHO geographic regions and contained ∼30,000 PSC and 27,000 WRA. Data were analyzed individually and combined with the use of a meta-analysis.

**Results:** The methods that were used to standardize the BRINDA database and the analytic approaches used to address the project’s research questions are presented in this article. Three approaches to adjust micronutrient biomarker concentrations in the presence of inflammation and malaria infection are presented, along with an anemia conceptual framework that guided the BRINDA project’s anemia analyses.

**Conclusions:** The BRINDA project refines approaches to interpret iron and vitamin A biomarker values in settings of inflammation and malaria infection and suggests the use of a new regression approach as well as proposes an anemia framework to which real-world data can be applied. Findings can inform guidelines and strategies to prevent and control micronutrient deficiencies and anemia globally.

## INTRODUCTION

Global and country-level decision makers frequently make policy and program decisions with the use of uncertain data. Improving methods to accurately estimate priority global health indicators facilitates the reduction of such uncertainty and assists policy makers in making better and more-informed decisions. In the nutrition field, there is a need for the accurate measurement of micronutrient biomarkers to determine public-health burdens and micronutrient-intervention priorities as well as to understand the relative contribution of micronutrient deficiencies to anemia. The presence of inflammation and infection pose problems with the interpretability of certain micronutrient biomarkers that can either be substantially overestimated or underestimated in the presence of inflammation and infections (1–3). Challenges exist in accurately assessing micronutrients and their role in anemia and in better understanding the other factors (infection, inflammation, and genetics) that contributed to anemia to formulate a response that matches the causes with the right interventions.

To this end, the Biomarkers Reflecting Inflammation and Nutritional Determinants of Anemia (BRINDA) project consists of a collaborative research group who used population-based nutrition surveys to address 3 primary research aims as follows: *1*) to assess factors that are associated with inflammation [defined as elevations in the acute phase proteins α-1-acid glycoprotein (AGP) and C-reactive protein (CRP)]; *2*) to assess the associations between AGP, CRP, and malaria infection with iron and vitamin A biomarkers and develop approaches to adjust for the effects of inflammation and malaria infection; and *3*) to identify risk factors and their relative contributions to anemia in preschool children (PSC) and women of reproductive age (WRA) across geographic settings.

An overview of the BRINDA project and the rational for our priority research questions have been described elsewhere (4). Briefly, the BRINDA project was formed in 2012 on the basis of an identified need to address programmatic issues related to inflammation, micronutrient biomarkers, and anemia etiology. The project is led by investigators from the *Eunice Kennedy Shriver* National Institute of Child Health and Human development of the NIH, the Global Alliance for Improved Nutrition, and the US CDC. A governing body was established to ensure project quality, shared ownership by partners, and proper management of project operations. The organizational structure includes an agency-led steering committee and investigator-led working groups. The investigators from the 3 lead agencies and representatives from the countries and agencies that contributed data carried out the research.

The present supplement consists of 9 articles including this methodologic overview article (**Table 1**). In this article, we lay the foundation for the approaches that were used in the subsequent articles. The first of the subsequent articles explores the demographic and health factors that are associated with inflammation (AGP and CRP) (5). The next 4 articles examine the influence of inflammation and malaria infection on iron and vitamin A biomarkers as well as potential methods for adjusting biomarker data to better account for the effects of inflammation (6–9). The following 2 articles assess the relations in iron deficiency, other risk factors, and anemia (10, 11). The final article highlights the research, policy, and programmatic considerations of the BRINDA project findings (12). To the best of our knowledge, the BRINDA project is the largest and most comprehensive project to date to use individual participant data to systematically address these research questions.

**TABLE 1:** Articles included in the BRINDA project supplement^1^

	Title
1	Methodologic approach for the BRINDA project
2	Factors associated with inflammation in preschool children and women of reproductive age: BRINDA project
3	Adjusting ferritin concentrations for inflammation: BRINDA project
4	Adjusting soluble transferrin receptor concentrations for inflammation: BRINDA project
5	Adjusting total body iron for inflammation: BRINDA project
6	Adjusting retinol-binding protein concentrations for inflammation: BRINDA project
7	Predictors of anemia in preschool children: BRINDA project
8	Predictors of anemia in women of reproductive age: BRINDA project
9	Research, policy, and programmatic considerations from the BRINDA project

1 BRINDA, Biomarkers Reflecting Inflammation and Nutritional Determinants of Anemia.

## METHODS

### BRINDA data-set selection

Data sets were selected on the basis of accessibility by the agencies leading the BRINDA project and partners. All data sets came from nationally or regionally representative population-based household-nutrition surveys that were conducted with similar sampling and data-collection methodologies. More information on the individual data sets that were used has been published elsewhere (13–25). The BRINDA project database inclusion criteria included surveys that *1*) were conducted after 2004; *2*) had target groups of PSC, WRA, or both; and *3*) used a similar laboratory methodology for the measurement of ≥1 biomarker of iron [ferritin or soluble transferrin receptor (sTfR)] or vitamin A status [retinol-binding protein (RBP) or retinol] and ≥1 biomarker of inflammation (AGP or CRP). Further information on the data-selection criteria is presented in the BRINDA project overview article (4). The study was reviewed by the Institutional Review Board of the NIH and was deemed to be non–human subjects research.

### BRINDA database management

A data management group (consisting of 6 members) was formed to generate a harmonized BRINDA project database that comprises data sets from multiple surveys. The following 3-step process was used to develop the database: central acquisition, conversion, and validation.

#### Central acquisition

The data management group obtained permission from the country survey implementer and organization to use their data. Once permission was obtained, a country survey representative was assigned to each data set. Country survey representatives were responsible for submitting data, supplementary materials, and a data intake form. The information provided included details on the survey design and methodology, quality assessment, and contextual information on the country’s nutrition and health interventions and disease burden at the time of the survey. Data from 17 surveys comprising 15 countries were obtained, and 16 surveys from 14 countries were ultimately used in the analysis (as described in Results). All data were de-identified before transfer to the BRINDA project and stored on a secure website.

#### Conversion

Two members of the data management group (OY Addo and A Williams) converted the individual data sets into a harmonized database with the use of SAS 9.4 software (SAS Institute). The harmonization process involved the identification of variables in the individual data sets that would be applicable to the BRINDA project’s research questions. Variable definitions were standardized with the use of a data dictionary that was developed for the project on the basis of commonalities in variable definitions across data sets. Anthropometric indexes were calculated with the use of variables that were obtained from the individual data sets with WHO software according to the 2006 WHO standard (WHO SAS Macro; WHO). Data-quality activities that were performed included range checking, logical checking, and structural checking at the time of data entry. Finally, individual data sets were merged into 2 master BRINDA project databases that were divided by target group (i.e., PSC and WRA).

#### Validation

One member of the data management group (SMLN) performed the validation of the harmonized database. This consisted of cross-checking the data-quality activities that were undertaken during the conversion stage as well as data-set consistency checks between the original data sets and the harmonized database. Discrepancies were shared with the full data management group to obtain agreement on modifications. If needed, survey representatives were recontacted to provide clarifications. Inconsistencies that could not be resolved by going back to the survey representatives were discussed in the data management group, and a decision by ≥3 members was taken as to how to proceed.

### BRINDA database laboratory analyses and variable definitions

Hemoglobin concentrations were measured with the use of a Beckman Coulter MAXM hematology flow cytometer (Beckman Coulter Inc.) in the United States or a portable hemoglobinometer in Georgia (HumaMeter; Human GmbH) and in all other countries (HemoCue; HemoCue AB) (**Table 2**). Blood samples from 9 of the data sets were transported to the VitMin Laboratory and AGP, CRP, ferritin, RBP, and sTfR were measured with the use of a sandwich ELISA (26). An additional data set (Papua New Guinea) measured AGP, CRP, sTfR, and RBP at the VitMin Laboratory from dried blood spots, but the same laboratory technique was applied. Ferritin values >80 μg/L were estimated by technicians at the VitMin Laboratory but were still retained in the data set. Samples from plasma using the anticoagulant EDTA may result in unstable sTfR results with prolonged exposure to room temperature and multiple freeze-thaw cycles (J Erhardt, VitMin Laboratory, personal communication, 2014). Of the samples that were sent to the VitMin Laboratory, 7 data sets used plasma containing EDTA, one data set used plasma containing heparin, and one data set used neither (dried blood spots). All samples were measured in duplicate in the VitMin Laboratory, and the intra-assay and interassay CVs were <10%. The VitMin Laboratory participates in the CDC Vitamin A Laboratory–External Quality Assurance interlaboratory comparison rounds and has a rigorous internal quality-control system. The use of the same multiplex assay to measure inflammation and micronutrient biomarkers increased the reliability and comparability of the results across data sets.

**TABLE 2:** Biomarker laboratory methods used in the 16 surveys and 14 countries composing the BRINDA project database^1^

Country (year) (reference)	AGP	CRP	Ferritin	Hemoglobin	RBP	sTfR	Retinol
Bangladesh (2010) (24)	Sandwich ELISA	Sandwich ELISA	Sandwich ELISA	Portable hemoglobinometer	Sandwich ELISA	Sandwich ELISA	NA
Cameroon (2009) (18)	Sandwich ELISA	Sandwich ELISA	Sandwich ELISA	Portable hemoglobinometer	Sandwich ELISA	Sandwich ELISA	HPLC
Colombia (2012) (23)	NA	Turbidimetry	Immunoassay	Portable hemoglobinometer	NA	NA	HPLC
Cote d'Ivoire (2007) (21, 22)	Sandwich ELISA	Sandwich ELISA	Sandwich ELISA	Portable hemoglobinometer	Sandwich ELISA	Sandwich ELISA	NA
Georgia (2009) (14)	NA	Turbidimetry	Immunoassay	Portable hemoglobinometer	NA	NA	NA
Kenya (2007, 2010) (17, 19)	Sandwich ELISA	Sandwich ELISA	Sandwich ELISA	Portable hemoglobinometer	Sandwich ELISA	Sandwich ELISA	NA
Laos (2006)	Sandwich ELISA	Sandwich ELISA	Sandwich ELISA	Portable hemoglobinometer	Sandwich ELISA	Sandwich ELISA	NA
Liberia (2011) (15)	Sandwich ELISA	Sandwich ELISA	Sandwich ELISA	Portable hemoglobinometer	Sandwich ELISA	Sandwich ELISA	NA
Mexico^2^ (2006, 2012) (23)	NA	Nephelometry	Immunoassay	Portable hemoglobinometer	NA	Immunoassay (2006)	HPLC (2012)
Nicaragua (2005) (20)	Turbidimetry	NA	Ramco ELISA	Portable hemoglobinometer	NA	NA	HPLC
Pakistan (2011) (16)	Immunoassay	NA	Immunoassay	Portable hemoglobinometer	NA	NA	HPLC
Philippines (2011) (25)	Sandwich ELISA	Sandwich ELISA	Sandwich ELISA	Portable hemoglobinometer	Sandwich ELISA	Sandwich ELISA	NA
PNG (2005) (13)	Sandwich ELISA	Sandwich ELISA	NA	Portable hemoglobinometer	Sandwich ELISA	Sandwich ELISA	NA
United States (2003–2006) (23)	NA	Immunoassay	Immunoassay	Hematology analyzer	NA	Immunoassay	HPLC

1 The VitMin Laboratory analyzed all samples in which the sandwich-ELISA technique was used. Hemoglobin concentrations were measured with Beckman Coulter MAXM hematology flow cytometer (Beckman Coulter Inc.) in the United States and with a portable hemoglobinometer in Georgia (HumaMeter; Human GmbH) and in all other countries (HemoCue; HemoCue AB). AGP, α-1-acid-glycoprotein; BRINDA, Biomarkers Reflecting Inflammation and Nutritional Determinants of Anemia; CRP, C-reactive protein; NA, not applicable; PNG, Papua New Guinea; RBP, retinol-binding protein; sTfR, soluble transferrin receptor.

2 sTfR was only available in the 2006 data set, and retinol was only available in the 2012 data set.

Seven data sets did not use the VitMin Laboratory to measure AGP, CRP, ferritin, RBP, or sTfR, although the assays and methods were all considered to be equivalent on the basis of previous validation testing of immunoassays compared with ELISA assays compared with HPLC between CDC and the VitMin Laboratory (R Whitehead, CDC International Micronutrient Malnutrition Prevention and Control program, personal communication, 2015) (Table 2). sTfR is typically measured with the use of either the Ramco ELISA or Roche assay. The interassay precision between the Ramco ELISA assay and the Roche assay is high, but the Roche assay measures much lower than the Ramco ELISA assay does (27). Therefore, sTfR values were multiplied by 1.6 in the data sets that used the Roche assay to allow for comparability (27). Retinol and RBP were used to define vitamin A deficiency (18). Although studies have shown retinol and RBP are closely correlated, the ratio of RBP:retinol may not always be 1:1 (18, 28). We had only one data set that measured both RBP and retinol, and thus, we could not further evaluate this relation and have assumed a ratio of 1:1 in our analyses as noted in the current WHO recommendations for retinol (29).

The same assays were not used across data sets; thus, there were varying limits of detection for certain biomarkers. A sensitivity analysis was conducted to assess issues with truncation and showed similar results when removing and including right- and left-truncated biomarker observations. Therefore, statistical methods to address the issue of truncation were not further explored (data not shown).

Five BRINDA data sets measured malaria infection. In the Kenya data sets and Côte d’Ivoire data set, malaria infection was assessed with the use of microscopy in which thick and thin smears were prepared with a single drop of blood, and malaria-infection slides were read by an experienced technician (30). The Paracheck Pf rapid diagnostic test (Orchid Biomedical System) was used in the Liberia data set (31), and plasma histidine-rich protein 2 (Cellabs Pty Ltd.) was used in the Cameroon data set (32). The standardization of malaria-infection diagnostic methods was explored, but because of the small difference in malaria-infection prevalence and the fact that, for the purposes of this research, malaria infection was used to make adjustments to micronutrient biomarkers and not to compare malaria-infection prevalence, standardization was not further pursued (33).

Biomarker thresholds were based on the WHO recommended cutoffs when available; when no such recommendations currently exist, we used thresholds that have been widely applied in the literature (**Table 3**).

**TABLE 3:** Cutoffs used for key biomarkers: the BRINDA project^1^

	Biomarker cutoff						
	AGP, g/L	CRP, mg/L	Ferritin, μg/L	Hemoglobin, g/L	RBP, μmol/L	sTfR, mg/L	Retinol, μmol/L
PSC	>1	>5	<12	Mild (<110)	<0.70	>8.3	<0.70
				Severe (<70)			
WRA	>1	>5	<15	Mild (<120)	<1.05	>8.3	<1.05
				Severe (<80)			
WHO recommended?	No	No	Yes	Yes	No	No	PSC: yes
							WRA: no
Reference	34	34, 35	36	37	18, 29, 38–40	41	29

1 AGP, α-1-acid-glycoprotein; BRINDA, Biomarkers Reflecting Inflammation and Nutritional Determinants of Anemia; CRP, C-reactive protein; PSC, preschool children; RBP, retinol-binding protein; sTfR, soluble transferrin receptor; WRA, women of reproductive age.

### Statistics

The analyses plans were developed with input from collaborating biostatisticians on the BRINDA project. Analyses were conducted at both the individual data-set level and the combined data-set level. Statistical survey packages (SAS 9.4; SAS Institute) survey procedures, STATA (version 12; StataCorp) svy command, and SPSS version 23 with the complex sample module (IBM Corp.) were used to account for complex survey designs that were used in each respective data set. In the analyses for the micronutrient biomarker–adjustments (6–8), individual data-set analyses that accounted for the complex survey design were done with the use of the survey package in R 3.2.2 software (R Core Team) (42). The individual data-set estimates were also combined with the use of the metafor package in R 3.2.2 software (R Core Team) (43). Heterogeneity of estimates across the data sets was assessed with the use of Cochran’s heterogeneity test (43, 44). In the analyses for inflammation (5) and anemia (10, 11) articles, heterogeneity of effects, both within and between data sets, was a concern. Thus, to combine the data sets and to examine heterogeneity, within–data-set weighting variables were rescaled so that the sum of the weights was proportional to the target population (PSC or WRA) of that survey.

#### Biomarker adjustments

The following 3 approaches are presented in this supplement to adjust ferritin, sTfR, and RBP biomarkers for CRP, AGP, malaria infection, or a combination: exclusion, correction factor (CF), and regression correction (RC) (**Table 4**). Prevalence estimates of micronutrient deficiencies with the use of no adjustment and the 3 adjustment approaches were compared with McNemar’s chi-square statistics. Statistical significance was defined as a *P* < 0.05 before applying the Bonferroni corrections to correct for multiple comparisons (*P* = 0.05 ÷ *k*, where *k* equals the number of comparisons).

**TABLE 4:** Approaches to adjust iron and vitamin A biomarkers for inflammation and malaria infection: the BRINDA project^1^

Approach	Method
Unadjusted	• No adjustments for AGP, CRP, or malaria infection.
Exclusion	• Exclude from the data set individuals with a CRP concentration >5 mg/L or AGP concentration >1 g/L or malaria-infection positive.
	• Calculate the estimated prevalence of micronutrient deficiency with the use of the remaining subsample.
CF	• Stratify the data set into groups by inflammation or malaria-infection status depending on the data availability and MB (types of categorizations listed below).
	• Calculate the CF (ratio of the MB value’s GM in the reference group to that of the respective inflammation or malaria-infection group) for each categorization with the use of the equation shown below.
	• Multiply the raw MB values by the appropriate group CF.
	Equation 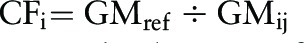
	where *i* denotes the data set, ref denotes the reference group, and *j* denotes the group.
	Category group:
	CRP: *1*) no inflammation (CRP concentration ≤5 mg/L) (reference); *2*) inflammation (CRP concentration >5 mg/L)
	AGP: *1*) no inflammation (AGP concentration ≤1 g/L) (reference); *2*) inflammation (AGP concentration >1 g/L)
	Malaria: *1*) malaria-infection negative (reference); *2*) malaria-infection positive.	
	CRP and AGP: *1*) no inflammation (CRP concentration ≤5 mg/L and AGP concentration ≤1 g/L) (reference); *2*) incubation (CRP concentration >5 mg/L and AGP concentration ≤1 g/L); *3*) early convalescence (CRP concentration >5 mg/L and AGP concentration >1 g/L); *4*) late convalescence (CRP concentration ≤5 mg/L and AGP concentration >1 g/L).
	AGP and malaria (sTfR only) (9): *1*) no inflammation and no malaria infection (AGP concentration ≤1 g/L and negative malaria infection); *2*) inflammation and no malaria infection (AGP concentration >1 g/L and malaria-infection negative); *3*) no inflammation and malaria infection (AGP concentration ≤1 g/L and positive malaria infection); and *4*) inflammation and malaria infection (AGP concentration >1 g/L and positive malaria infection).
RC	• Run linear regression models; outcome variable is ln MB; depending on available data, ln CRP and ln AGP (continuous) and malaria infection (dichotomous) can be included in the model as explanatory variables.
	• Extract slopes from explanatory variables and input into RC equation shown below (slope values multiplied by the CRP, AGP, and malaria infection observations and subtracted from the MB observations).
	• Back-transform adjusted MB values before applying MB cutoffs.
	Equation: 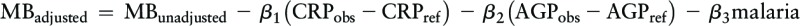
	where β_1_ is the CRP regression coefficient, β_2_ is the AGP regression coefficient, β_3_ is the malaria regression coefficient, obs denotes the observed value, and ref denotes the reference value. MBs CRP, AGP, CRP_ref_, and AGP_ref_ are on the ln scale; refs are the maximum values of the lowest CRP and AGP deciles obtained from the combined BRINDA database. The unlogged reference concentrations are as follows—CRP in PSC: 0.10 mg/L; CRP in WRA: 0.16 mg/L; AGP in PSC = 0.59 g/L; and AGP in WRA = 0.53 g/L; only apply adjustments to individuals with either CRP concentrations > CRP_ref_, AGP concentrations > AGP_ref_, or both.		

1 sTfR was adjusted for AGP and malaria infection but not for CRP per a biological rationale as described elsewhere (9). AGP, α-1-acid-glycoprotein; BRINDA, Biomarkers Reflecting Inflammation and Nutritional Determinants of Anemia; CF, correction factor; CRP, C-reactive protein; GM, geometric mean; MB, micronutrient biomarker; PSC, preschool children; RC, regression correction; sTfR, soluble transferrin receptor; WRA, women of reproductive age.

#### Categorical adjustment

We made categorical adjustments to the micronutrient biomarkers for inflammation (CRP concentration >5 mg/L, AGP concentration >1 g/L), malaria (positive infection), or a combination with the use of the exclusion and CF approaches. The exclusion approach entailed estimating the micronutrient-deficiency prevalence with the use of the subsample of the data set in which individuals had nonelevated inflammation or no malaria infection (Table 4). The CF approach involved applying CFs to the raw micronutrient biomarker values with the use of a CF equation as previously described (34, 45) (Table 4). Under this approach, the micronutrient values of individuals who were classified as having inflammation (CRP concentration >5 mg/L or AGP concentration >1 g/L) or malaria (positive infection) were shifted to a reference group (i.e., the segment of the population with no inflammation or malaria infection) (45).

CFs were generated in 3 ways as follows: with internal survey specific data (termed internal correction factor), a previous meta-analysis with the use of distinct data from the BRINDA database (termed Thurnham correction factor) (34, 46), or the meta-analyzed BRINDA database (termed BRINDA correction factor). The BRINDA correction factor was calculated by obtaining coefficient estimates and their variance-covariance matrix from the 2- and 4-group linear regression models from individual data sets and using these values in 2- and 4-group meta-analyses to calculate the combined effect sizes with the use of multivariable random-effects models to allow for variations between data sets. The meta-analyses were conducted on the ln scale, and the synthesized estimates were exponentiated to obtain the multiplicative CFs for the micronutrient biomarker adjustment. Sample sizes were used as weights to combine the summary statistics. The restricted maximum-likelihood approach with an unstructured variance-covariance matrix was used for the meta-analysis.

#### Continuous adjustment

We made linear adjustments to the micronutrient biomarkers for inflammation (CRP and AGP on a continuous scale) and a categorical adjustment for malaria (positive infection) with the use of the RC approach. Under this approach, we developed a regression-adjustment equation, which consisted of CRP or AGP slopes, or slopes for both (with or without a malaria-infection slope) from a linear regression model and an external reference value (Table 4).

The first step was to build the linear regression model (with the micronutrient biomarker as the outcome and CRP, AGP, malaria infection or a combination, as the explanatory variables). We performed regression diagnostics on the model to examine the appropriateness of the linearity and other modeling assumptions. Box-Cox transformations were used to determine the optimal transformation of the outcome variables (ferritin, sTfR, and RBP) (47). On the basis of this procedure, the ln transformation was deemed most appropriate for ferritin, sTfR, and RBP. Residuals, leverage, and influence were assessed for each data set with the use of the svydiags package in R 3.2.2 software (48, 49). An examination of the residual plot, scatter plots, and Cook’s *D* resulted in the decision to also use the ln for the explanatory variables CRP and AGP. Collinearity was also assessed in multiple linear regression models that included both CRP and AGP (including or excluding malaria infection) on the basis of tolerance >0.1 and <1 and a variance inflation factor <5.

The second component of the regression-adjustment equation was to include a reference value for CRP and AGP to avoid overadjusting for low levels of inflammation. A common definition of low levels of inflammation across data sets and laboratory methods was obtained with the use of a combined lowest decile (10th percentile) of CRP and AGP from each data set. The deciles were combined with the use of random-effects meta-analysis whereby the metaphor package in R 3.2.2 software was used to obtain a common estimate across data sets [termed external decile (ED)].

The ED was derived with the use of the full BRINDA database with the exception of data from Georgia because the lower concentration of CRP detection was too high (0.5 mg/L). The EDs that were estimated for the regression approach were as follows: CRP = −2.26 ln(mg/L) and AGP = −0.52 ln(g/L) in PSC; and CRP = −1.83 ln(mg/L) and AGP = −0.63 ln(g/L) in WRA. These EDs equate to a CRP concentration of 0.10 mg/L and AGP concentration of 0.59 g/L in PSC and a CRP concentration of 0.16 mg/L and AGP concentration of 0.53 g/L in WRA. A heterogeneity test for the ED was calculated although Mexico 2006 and United States data sets were excluded from the test because these data sets had zero variance in the lowest decile. There was some degree of heterogeneity, but we conducted a sensitivity analysis, and there were no meaningful differences in micronutrient-deficiency prevalences of the regression-adjusted micronutrient biomarkers with the use of internally generated deciles than with EDs (data not shown).

RCs were generated with the use of internal survey-specific data (termed internal regression correction). An SAS macro to adjust micronutrient biomarkers with the use of the internal regression correction approach is shown in the **Supplemental Materials**. In addition, external RCs were derived with the use of the meta-analyzed BRINDA database (termed BRINDA regression correction). The BRINDA regression correction was generated by running a linear regression to calculate the coefficients for each individual data set and running a meta-analysis to compute a mean coefficient.

#### Factors associated with inflammation and risk factors for anemia

To quantify the potential factors associated with inflammation, bivariable and multivariable logistic regression models were estimated with inflammation as the outcome with the use of clinically relevant cutoffs. The inflammation article (5) used an exploratory approach to quantify the potential factors associated with inflammation. Variables were selected for inclusion in a multivariable model if they were significant in the bivariable models by calculating Wald’s chi-square test. Data were analyzed at the individual data-set level. In addition, a pooled analyses restricted to countries with malaria data was conducted to further investigate the role of malaria in inflammation when controlling for sociodemographics and anthropometric variables.

The anemia articles (10, 11) used a conceptual approach to quantify the potential factors associated with anemia because the evidence base is better established than that for inflammation. Bivariable and multivariable models were estimated with anemia and severe anemia as the outcomes with the use of clinically relevant cutoffs both separately for each data set as well as pooled by countries with low, medium, and high burdens of infectious disease. The modeling strategy adopted was the “block stepwise regression approach” (50). Variables that have been considered in the literature to have high theoretical importance were retained in the model irrespective of significance. The other variables were examined in blocks starting with the proximal variables in the causal pathway and ending with distal variables. Theoretically, plausible 2-way interactions that were based on theory were tested and remained in the model at the *P* ≤ 0.10 level of significance.

The articles on potential factors associated with inflammation (5) and risk factors for anemia (10, 11) used statistical approaches to adjust for the sampling weight, strata, and cluster (as applicable) to account for the complex survey design for an individual data-set analysis. For pooled analyses, population-based weights were converted to standardized weights by dividing the individual weights by the mean population-based weight. The sum of the study-level weights was rescaled to represent the size of the population represented by the data set. In the multivariable analysis, variables that were not measured in ≥3 data sets were omitted.

## RESULTS

### Data characteristics

**FIGURE 1:**
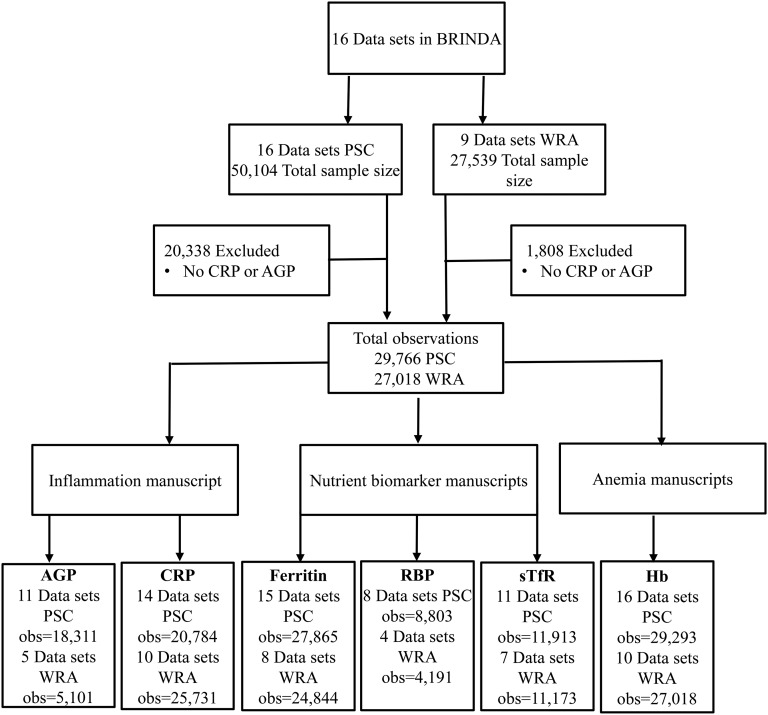
Sample size for BRINDA project analyses. The total sample size for BRINDA analyses ranged from 8803 to 29,293 for PSC and from 4191 to 27,018 for WRA. The total AGP sample size ranged from 5101 (WRA) to 18,311 (PSC); the CRP sample size ranged from 20,784 (PSC) to 25,731 (WRA); the ferritin sample size ranged from 24,844 (WRA) to 27,865 (PSC); the RBP sample size ranged from 4191 (WRA) to 8803 (PSC); the sTfR sample size ranged from 11,173 (WRA) to 11,913 (PSC); the anemia sample size ranged from 27,018 (WRA) to 29,293 (PSC). AGP, α-1-acid-glycoprotein; BRINDA, Biomarkers Reflecting Nutritional Determinants of Anemia; CRP, C-reactive protein; Hb, hemoglobin; obs, observed values; PSC, preschool children; RBP, retinol-binding protein; sTfR, soluble transferrin receptor; WRA, women of reproductive age.

A total of 23 data sets were initially identified; 2 data sets did not meet the study criteria, 2 studies did not have data ready, 2 data sets did not obtain appropriate permission, and 1 data set (Oman) (51) was removed because the laboratory methods that were used to measure CRP could not be validated against the other BRINDA data sets. With the final set of 16 data sets from 14 countries, the database collectively covered all 6 WHO geographic regions (52) with pooled sample sizes of *n* = 29,766 PSC and *n* = 27,018 WRA (**Figure 1**). The demographic and biomarker data that were available in the individual data sets varied considerably (**Table 5**). The key biomarkers that were used in this supplement were as follows: AGP (*n* = 11 PSC and 5 WRA data sets), anemia (*n* = 16 PSC and 10 WRA data sets), body iron (*n* = 8 PSC and 4 WRA data sets), CRP (*n* = 14 PSC and 10 WRA data sets), ferritin (*n* = 15 PSC and 8 WRA data sets), malaria infection (*n* = 5 PSC and 3 WRA data sets), RBP (*n* = 8 PSC and 4 WRA data sets), and sTfR (*n* = 11 PSC and 7 WRA data sets). Retinol was measured in 5 PSC and 2 WRA data sets and was not included in the biomarker articles because only one country measured both CRP and AGP. The retinol data were included in the anemia articles.

**TABLE 5:** Variables included in the harmonized database in preschool children and women of reproductive age: the BRINDA project^1^

	*n*	
Variable	PSC^2^	WRA^3^	Bangladesh	Cameroon	CI	Colombia	Georgia	Kenya	Laos	Liberia	Nicaragua	Mexico^4^	Pakistan	Philippines	PNG	United States
Education																
Household	15,364	11,748	—	X	—	X	—	—	X	—	—	X	C	—	—	X
Maternal	15,170	NA	—	C	C	C	—	C	C	—	C	—	C	C	—	—
Respondent	13,259	—	—	W	W	W	W	—	W	W	—	—	—	—	W	W
SES	26,723	25,535	—	X	X	X	X	C	X	X	—	X	C	C	X	X
Water	18,007	7736	C	X	X	X	—	C	X	X	C	C	C	C	C	—
Sanitation	19,525	10,813	—	X	X	X	—	C	X	X	—	X	—	—	C	—
Vitamin A use	13,799	—	C	C	C	—	—	C	C	C	C	C	C	C	C	—
Iron use	3856	2602	C	X	—	—	—	C	—	W	—	—	—	—	—	—
Multivitamin use	13,509	1464	—	X	X	—	—	C	—	X	—	—	C	C	—	—
Recent illness																
Cough	18,249	7497	—	—	X	—	C	—	C	C	—	X	C	C	—	—
Diarrhea	19,917	7497	—	—	X	—	C	C	C	C	—	X	C	C	—	—
Fever	15,810	834	—	—	X	—	C	C	C	C	—	—	C	C	—	—
Respiratory	12,539	6663	—	—	—	—	C	—	—	—	—	X	C	—	—	—
Anthropometric measures	29,436	23,416	C	X	X	X	X	C	X	C	C	X	C	C	X	X
Hemoglobin	29,293	25,673	C	X	X	X	X	C	X	X	C	X	C	C	X	X
Micronutrients																
Ferritin	27,911	24,938	C	X	X	X	X	C	X	X	C	X	C	C	—	X
Folate	4703	7992	—	—	W	—	W	—	—	—	—	W	—	—	—	X
RBC folate	1299	3178	—	—	—	—	—	—	—	—	—	—	—	—	—	X
RBP	8849	4285	C	X	X	—	—	C	—	X	—	—	—	C	X	—
Retinol	15,163	3249	—	X^5^	—	C	—	—	—	—	C	C	C	—	—	X
sTfR	11,960	11,267	C	X	X	—	—	C	X	X	—	X	—	C	X	X
Iodine	NA	2538	—	—	—	—	—	—	W	—	—	—	—	—	W	W
Vitamin B-12	4781	7784	—	—	X	X	—	—	—	—	—	W	—	—	—	X
Vitamin B-6	479	1531	—	—	—	—	—	—	—	—	—	—	—	—	—	X
Vitamin D	8257	3196	—	—	—	—	—	—	—	—	—	—	C	—	—	X
Zinc	12,267	—	—	—	—	C	—	—	—	—	—	C	C	—	—	W
ZPP	—	1823	—	—	W	—	—	C	—	—	—	—	—	—	—	—
Inflammation																
AGP	18,311	—	C	X	X	—	—	C	X	X	C	—	C	C	X	—
CRP	20,784	25,731	C	X	X	X	X	C	X	X	—	X	—	C	X	X
Malaria infection	4672	3442	—	X	X	—	—	C	—	X	—	—	—	—	—	—
Helminths	272	—	—	—	—	—	—	—	C	—	—	—	—	—	—	—
Hemoglobinopathies	856	NA	—	—	—	—	—	C^6^	—	—	—	—	—	—	—	—

1 AGP, α-1-acid-glycoprotein; BRINDA, Biomarkers Reflecting Inflammation and Nutritional Determinants of Anemia; C, available in preschool children only; CI, Cote d’Ivoire; CRP, C-reactive protein; NA, not applicable; PNG, Papua New Guinea; PSC, preschool children; RBC, red blood cell; RBP, retinol-binding protein; SES, socioeconomic status; sTfR, soluble transferrin receptor; W, available in women of reproductive age only; WRA, women of reproductive age; X, available in preschool children and women of reproductive age; ZPP, zinc protoporphyrin.

2 Age range per data set was as follows: Bangladesh 2010 (6–11 mo), Cameroon 2009 (12–59 mo), Colombia 2010 (6–59 mo), Cote d'Ivoire 2007 (6–59 mo), Georgia 2009 (12–59 mo), Kenya 2007 and 2010 (6–35 mo), Laos 2006 (6–59 mo), Liberia 2011 (6–35 mo), Mexico 2006 and 2012 (12–59 mo), Nicaragua 2005 (6–59 mo), Pakistan 2011 (6–59 mo), Philippines 2011 (6–23 mo), PNG 2005 (6–59 mo), and United States 2003–2006 (6–59 mo).

3 Age range per data set was 15–49 y.

4 Water, sanitation, retinol, sTfR, and zinc were only measured in the Mexico 2006 data set; folate (WRA) and vitamin B-12 (WRA) were only measured in the Mexico 2012 data set.

5 Only measured in a subset of the data set.

6 Data were only measured in Kenya 2010 data set.

### Data quality

Blood specimens were hemolyzed in <5% of samples in 7 data sets and between 5% and 10% in 3 data sets (information unavailable in *n* = 6 data sets). A subsample of the laboratory tests was sent for external quality control in 7 of 14 data sets (information unavailable in *n* = 2 data sets).

### Categorical adjustments to micronutrient biomarkers

Combined mean concentrations of ferritin, sTfR, and RBP across data sets at the different stages of the acute-phase protein response (incubation, early convalescence, and late convalescence) are presented in **Table 6**. Mean ferritin concentrations were lowest in the reference group and were highest in those in the early convalescence stage in PSC and WRA, whereas the reverse association was the case for RBP in PSC and WRA. The lowest mean sTfR concentration was in the incubation group, and the highest mean sTfR concentration was in the early convalescence group in PSC and WRA.

**TABLE 6:** Pooled ferritin, sTfR, and RBP in preschool children and women of reproductive age according to inflammation stage: the BRINDA project^1^

Inflammatory stage	*n*	Ferritin, μg/L	*n*	sTfR, mg/L	*n*	RBP, μmol/L
Preschool children						
Reference	4400	19.50 (14.91, 25.51)^2^	4766	7.37 (5.78, 9.40)	4495	0.98 (0.94, 1.02)
Incubation	280	28.48 (21.16, 38.33)	306	6.96 (5.36, 9.05)	293	0.80 (0.76, 0,85)
Early convalescence	1771	50.79 (40.54, 63.62)	2028	8.58 (6.59, 11.17)	1965	0.71 (0.68, 0.74)
Late convalescence	1962	29.79 (23.68, 37.48)	2181	8.45 (6.53, 10.94)	2050	0.90 (0.87, 0.93)
Women of reproductive age						
Reference	3366	32.04 (28.60, 35.89)	3927	6.15 (4.51, 8.40)	3232	1.43 (1.32, 1.56)
Incubation	342	48.48 (38.16, 61.58)	365	5.85 (3.95, 8.67)	329	1.27 (1.18, 1.37)
Early convalescence	285	61.67 (53.82, 70.67)	338	7.29 (5.76, 9.23)	310	1.19 (1.07, 1.34)
Late convalescence	265	38.94 (30.92, 49.03)	374	7.13 (5.58, 9.10)	320	1.53 (1.43, 1.63)

1 Reference is defined as a CRP concentration ≤5 mg/L and AGP concentration ≤1 g/L; incubation is defined as a CRP concentration >5 mg/L and AGP concentration ≤1 g/L; early convalescence is defined as a CRP concentration >5 mg/L and AGP concentration>1 g/L; and late convalescence is defined as an AGP concentration >1 g/L and CRP concentration ≤5 mg/L. Data sets had to have a biomarker for CRP and AGP to be included. Ferritin: PSC QE (df = 28) = 578.0 (*P* < 0.0001) and WRA QE (df = 12) = 42.1 (*P* < 0.0001); sTfR: PSC QE (df = 32) = 3015.6 (*P* < 0.0001) and WRA QE (df = 16) = 897.0 (*P* < 0.0001); and RBP: PSC QE (df = 28) = 250.6 (*P* < 0.0001) and WRA QE (df = 12) = 120.6 (*P* < 0.0001). AGP, α-1-acid-glycoprotein; BRINDA, Biomarkers Reflecting Inflammation and Nutritional Determinants of Anemia; CRP, C-reactive protein; PSC, preschool children; RBP, retinol-binding protein; sTfR, soluble transferrin receptor; WRA, women of reproductive age.

2 Geometric mean; 95% CI in parentheses (all such values).

**FIGURE 2:**
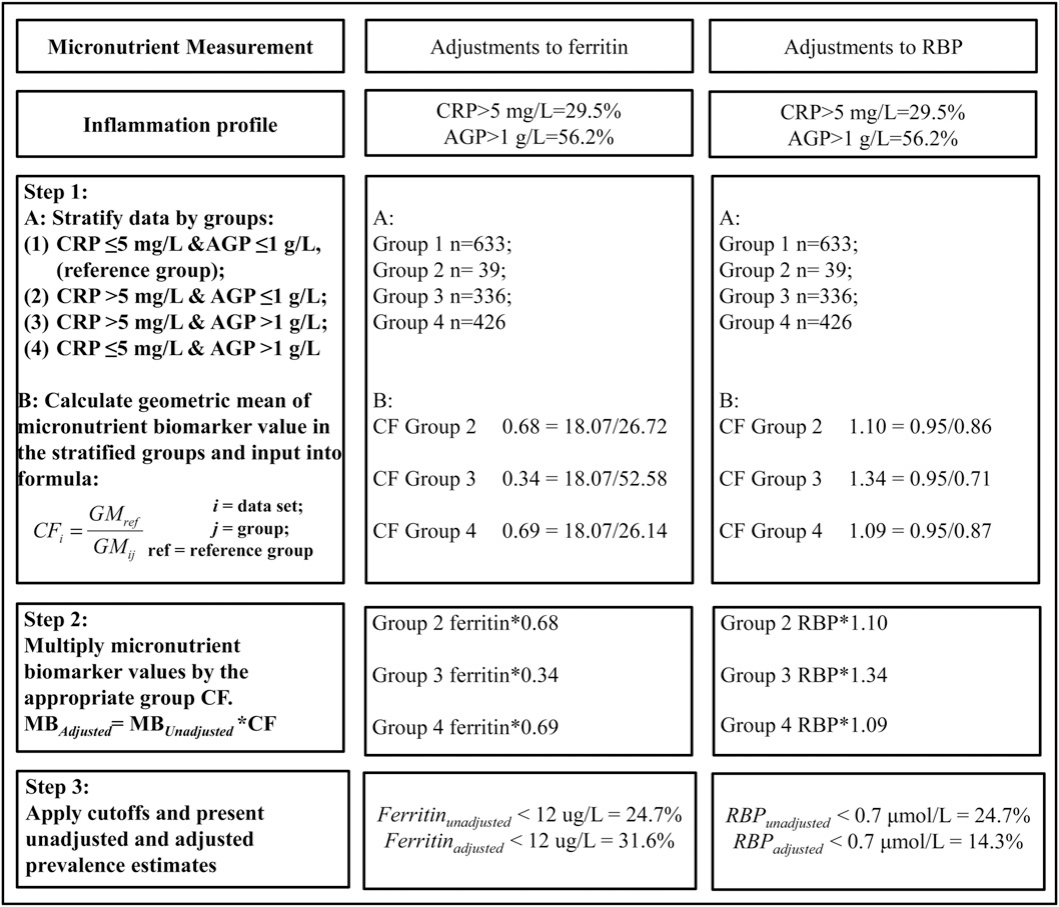
Adjustments to ferritin and RBP with the use of the internal CF approach: an illustrative example in preschool in Liberia: Biomarkers Reflecting Nutritional Determinants of Anemia (BRINDA) project. AGP, α-1-acid-glycoprotein; CF, correction factor; CRP, C-reactive protein; GM, geometric mean; MB, micronutrient biomarker; RBP, retinol-binding protein.

**Figure 2** provides an illustrative example of how to adjust an iron biomarker (ferritin) and vitamin A biomarker (RBP) for inflammation with the use of the CF approach on the basis of real data from Liberia in PSC. The application of a ferritin-concentration cutoff <12 μg/L to the unadjusted and adjusted ferritin concentrations resulted in an estimated unadjusted prevalence of depleted iron stores of 20.4% compared with an adjusted prevalence of 31.6%. The application of an RBP-concentration cutoff <0.7 μmol/L to the unadjusted and adjusted RBP concentrations resulted in an estimated unadjusted prevalence of vitamin A deficiency of 24.7% compared with an adjusted prevalence of 14.3%.

### Continuous adjustments to micronutrient biomarkers

**FIGURE 3:**
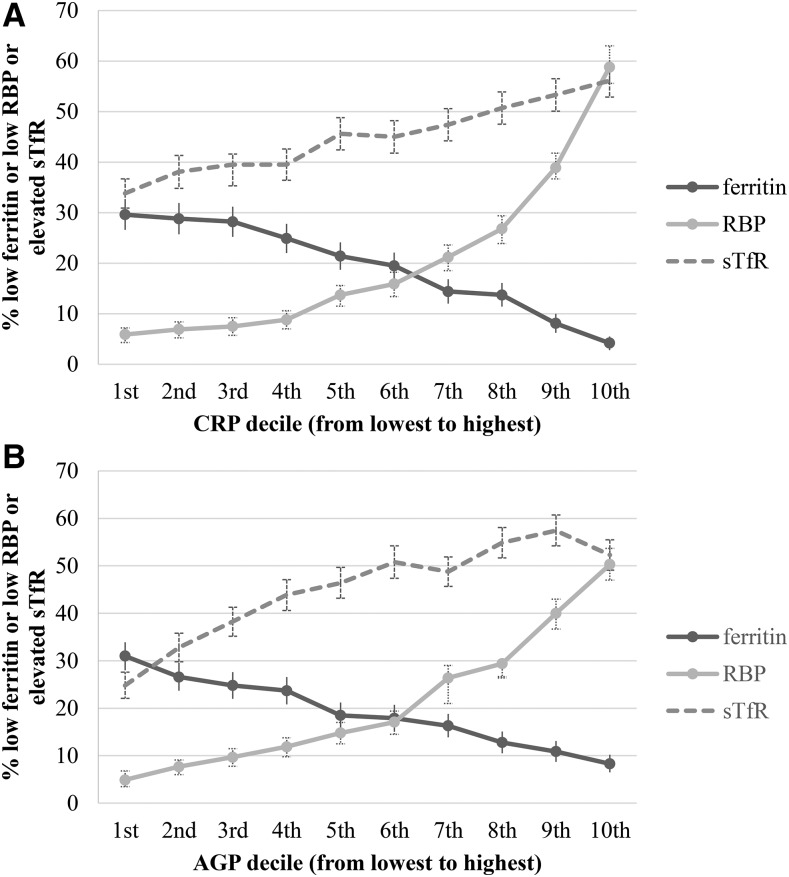
Depleted iron stores, vitamin A deficiency, and iron-deficient erythropoiesis [percentages (95% CI )] in preschool children by CRP and AGP deciles: Biomarkers Reflecting Nutritional Determinants of Anemia (BRINDA) project. Prevalences of low ferritin concentrations (<12 μg/L), low RBP concentrations (<0.70 μmol/L), and elevated sTfR concentrations (>8.3 mg/L) are stratified by CRP decile (A) and AGP decile (B). The analysis was restricted to data sets [Bangladesh, Cameroon, Côte d’Ivoire, Kenya 2007, Kenya 2010, Laos, Liberia, Philippines, and Papua New Guinea (except there were no data on ferritin in Papua New Guinea)] that measured both CRP and AGP for comparability between CRP and AGP relations with biomarkers. Ferritin: *n* = 8458; RBP: *n* = 8848; and sTfR: *n* = 9326. AGP, α-1-acid-glycoprotein; CRP, C-reactive protein; RBP, retinol-binding protein; sTfR, soluble transferrin receptor.

A linear trend between the inflammation decile and the micronutrient biomarkers is shown in **Figures 3** (PSC) and **4** (WRA). In PSC, depleted iron stores (ferritin concentration <12 μg/L) in the lowest-inflammation group was higher than in the highest-inflammation group, and, as expected, the reverse relation was shown for the prevalence of iron-deficient erythropoiesis (sTfR concentration >8.3 mg/L) and vitamin A deficiency (RBP concentration >0.70 μmol/L) in PSC. The linearity and the magnitude of the relation between inflammation deciles and micronutrient biomarkers was dampened in WRA compared with in PSC especially in the case of vitamin A deficiency.

**FIGURE 4:**
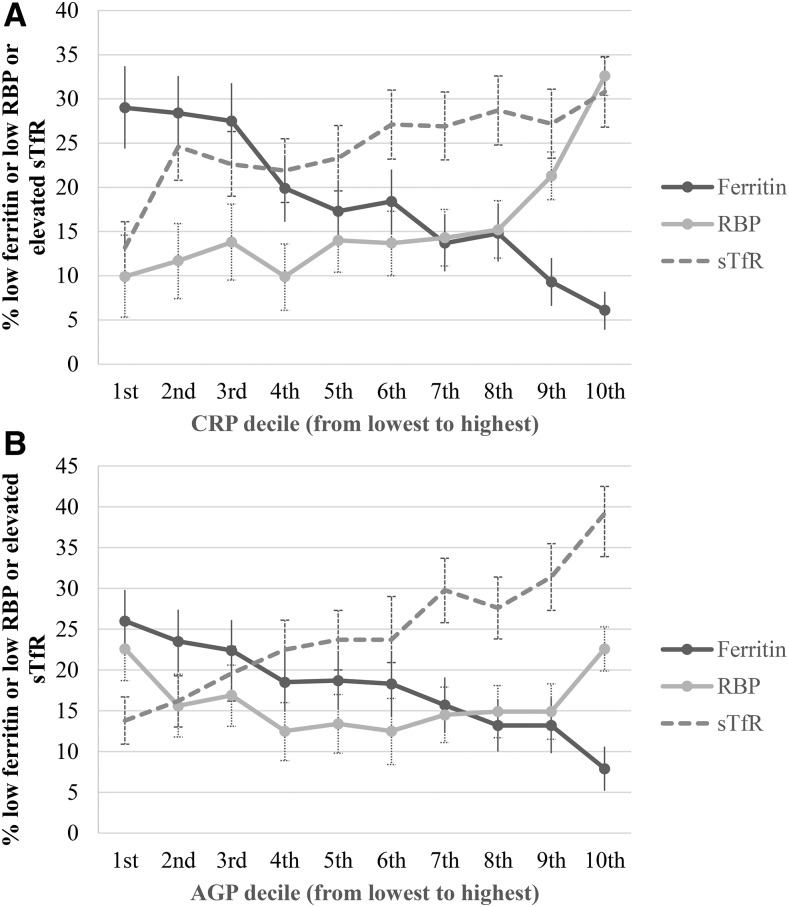
Depleted iron stores, vitamin A deficiency, and iron-deficient erythropoiesis [percentages (95% CIs)] in women of reproductive age by CRP and AGP deciles: Biomarkers Reflecting Nutritional Determinants of Anemia (BRINDA) project. Prevalences of low ferritin concentrations (<15 μg/L), low RBP concentrations (<1.05 μmol/L), and elevated sTfR concentrations (>8.3 mg/L) are stratified by CRP decile (A) and AGP decile (B). The analysis was restricted to data sets [Cameroon, Côte d’Ivoire, Laos (except there were no data on RBP in Laos), Liberia, and Papua New Guinea (except there were no data on ferritin in Papua New Guinea)] that measured both CRP and AGP for comparability between CRP and AGP relations with biomarkers. Ferritin: *n* = 4352; RBP: *n* = 4285; and sTfR: *n* = 5098. AGP, α-1-acid-glycoprotein; CRP, C-reactive protein; RBP, retinol-binding protein; sTfR, soluble transferrin receptor.

**FIGURE 5:**
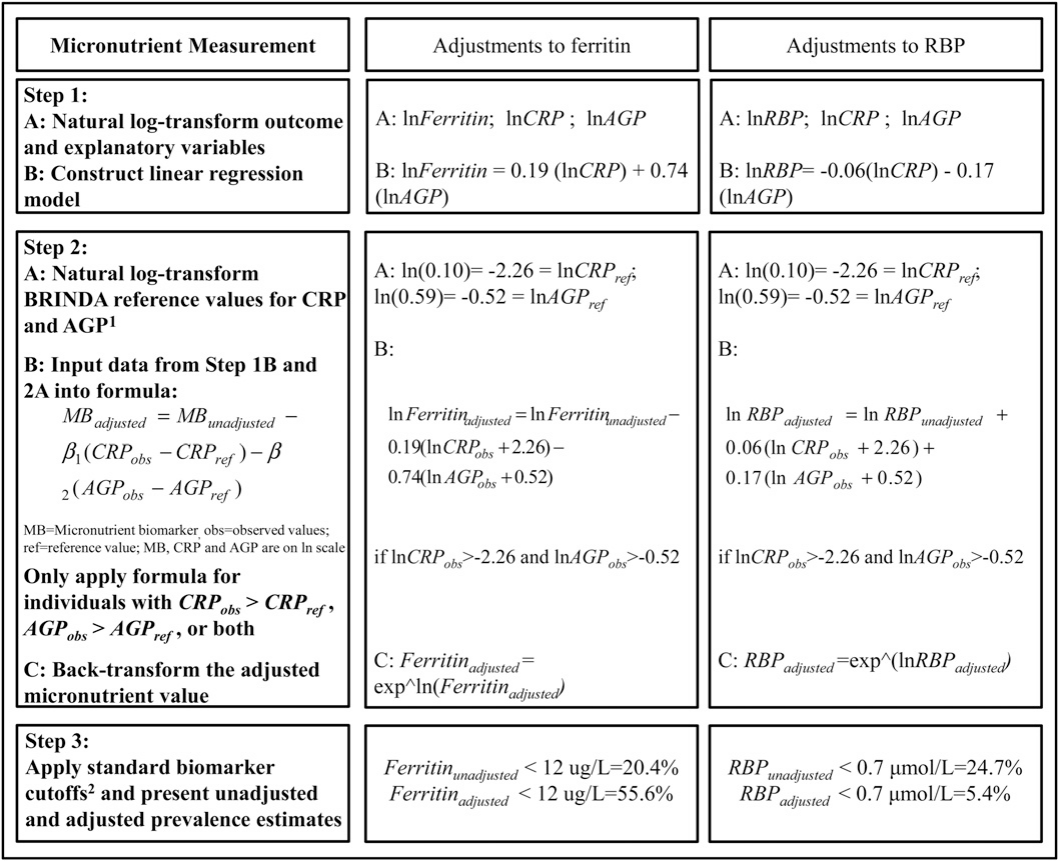
Adjustments to ferritin and RBP for inflammation (CRP and AGP) with the use of the internal regression correction approach: an illustrative example in preschool children in Liberia. ^1^Nonlogged BRINDA reference values using external deciles were a CRP concentration of 0.10 mg/L and AGP concentration of 0.59 g/L in preschool children and a CRP concentration of 0.16 mg/L and AGP concentration of 0.53 g/L in women of reproductive age. ^2^MB cutoffs are listed in Table 3. AGP, α-1-acid-glycoprotein; BRINDA, Biomarkers Reflecting Inflammation and Nutritional Determinants of Anemia; CRP, C-reactive protein; MB, micronutrient biomarker; RBP, retinol-binding protein.

**Figure 5** provides an illustrative example of how to adjust an iron biomarker (ferritin) and vitamin A biomarker (RBP) for inflammation with the use of the internal regression correction approach on the basis of real data from Liberia in PSC. The application of a ferritin-concentration cutoff <12 μg/L to the unadjusted and adjusted ferritin concentrations resulted in an estimated unadjusted prevalence of depleted iron stores of 20.4% compared with an adjusted prevalence of 55.6%. The application of an RBP-concentration cutoff <0.7 μmol/L to the unadjusted and adjusted RBP concentrations resulted in an estimated unadjusted prevalence of vitamin A deficiency of 24.7% compared with an adjusted prevalence of 5.4%.

### Factors associated with inflammation and risk factors for anemia

The authors of the article that examined factors associated with inflammation (5) used exploratory modeling. One reason for conducting this analysis was to determine whether there were additional variables that should be considered when adjusting micronutrient biomarkers. There was limited data on infections besides malaria, and there was variability in both the availability and definition of self-reported morbidity (proxy for infections) in the data sets within the BRINDA database. Furthermore, the authors showed that the relation between self-reported morbidity (proxy for infections) and inflammation was inconsistent. For this reason, adjustments for infections and self-reported morbidity were not further explored in the biomarker-adjustment papers (6–9).

**FIGURE 6:**
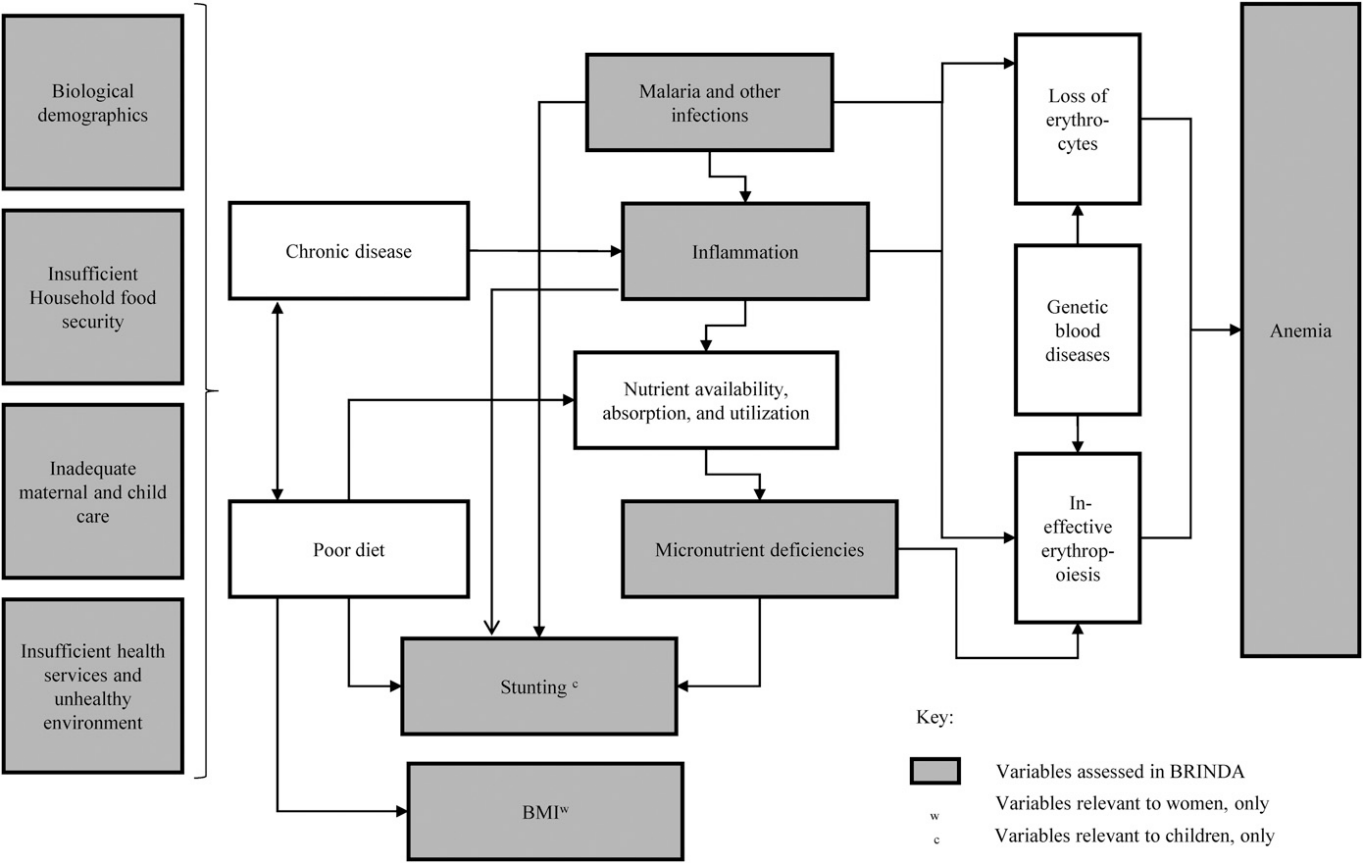
The BRINDA project's Anemia conceptual framework showing variables included in the BRINDA database. BRINDA, Biomarkers Reflecting Inflammation and Nutritional Determinants of Anemia.

In contrast, the authors of the articles examining factors associated with anemia (10, 11) developed an anemia conceptual framework to guide the modeling process because the theoretical relation between anemia and its risk factors is well established. The anemia framework is applicable to PSC and WRA and is presented here to lay the foundation for the anemia articles (**Figure 6**). Going from left to right in Figure 6, the framework shows the underlying and immediate factors that lead to the loss of erythrocytes and ineffective erythropoiesis and ultimately result in anemia. The underlying causes of anemia were adopted from the well-established UNICEF malnutrition framework (household food insecurity, inadequate care, unhealthy household environment, and lack of health services) (53). The framework further illustrates a causal chain of events whereby chronic disease and poor diet increase risks of malaria infection, other infections, inflammation, nutritional deficiencies, and a combination (either synergistic or antagonistic) of these factors, which ultimately lead to anemia. Stunting has been included as a correlate with anemia because the 2 phenomena frequently overlap although this relation may not be causal in nature.

## DISCUSSION

The first important contribution of the BRINDA project was to refine approaches to estimate the prevalence of micronutrient deficiencies (6–8). The BRINDA group compared different approaches to account for the effects of inflammation and malaria infection on iron and vitamin A biomarkers with the use of a predefined set of characteristics to summarize the strengths and weaknesses of each approach (**Table 7**). The BRINDA group was only able to consider 3 of 5 characteristics as follows: *1*) precision, *2*) variability reflecting relation between micronutrient biomarkers and severity of inflammation, and *3*) feasibility to implement across countries. Two other important considerations (validity and the ability to monitor trends) could not be assessed with the use of the current BRINDA database because gold-standard micronutrient biomarkers and time-series data were not available. These characteristics should be assessed in future studies.

**TABLE 7:** Suggested criteria to compare biomarker-adjustment approaches: the BRINDA project^1^

	Exclusion	Internal correction factor	Thurnham/BRINDA correction factor	Internal regression correction	BRINDA regression correction
Precision^2^	+	+	++	++	+++
Variability reflecting the relation between micronutrient biomarkers and severity of inflammation	+	+	+	+++	?
Feasibility	+++	++	+++	+	+
Validity^3^	?	?	?	?	?
Monitor trends^4^	?	?	?	?	?

1 BRINDA, Biomarkers Reflecting Inflammation and Nutritional Determinants of Anemia; +, low; ++, medium; +++, high; ?, unable to assess.

2 Precision (as dictated by the sample size) is expected to be lower with the use of exclusion and internal correction factor approaches than with the use of the Thurnham/BRINDA correction factor approach because the sample size in the former approaches is based on the subset of the population without inflammation in an individual data set, whereas the latter approach is based on a larger external data set. Similarly, the precision is likely lower for the internal regression correction approach than for the BRINDA regression correction. However, the heterogeneity of the individual country adjustments may affect the overall precision of the Thurnham/BRINDA correction factor and BRINDA regression correction approaches.

3 Could not be assessed without a gold standard.

4 Data were derived from cross-sectional surveys; only 2 countries had time-series data, and trends were not assessed.

All adjustment approaches resulted in substantial changes in the estimated prevalence of micronutrient deficiencies compared with unadjusted values with even greater differences when the RC approach was used. The CF approach resulted in similar prevalence estimates as with the exclusion approach but had the advantage of retaining the full sample. The exclusion and CF approaches rely on cutoffs for elevated CRP and AGP that were not derived for the purposes of adjusting micronutrient biomarkers; thus, the relevance of these cutoffs when adjusting for micronutrient biomarker concentrations is unclear. The BRINDA group concluded that the RC approach was more promising than the exclusion or CF approach was because it accounted for the influence of the entire spectrum of inflammation. With the use of the BRINDA database, we illustrated that there was no clear threshold value below which the micronutrient biomarkers (ferritin, sTfR, and RBP) were unaffected by CRP and AGP; rather, the relation appeared to follow a linear pattern throughout the range of CRP and AGP, thereby lending support for the use of the RC approach.

Other authors have used the RC approach (18, 54), but the reference value that was selected by the BRINDA group to avoid overadjustment at the lower concentrations of AGP and CRP was a major divergence from previous studies. For instance, Engle-Stone et al. (18) used a similar RC approach but selected higher reference values of 3.75 mg/L for the CRP concentration and 0.75 g/L for the AGP concentration. In addition, the authors only applied the RC adjustments to those individuals with CRP concentrations >5 mg/L or AGP concentration >1 g/L. The BRINDA group showed that even at low values of CRP and AGP there was an effect on micronutrient biomarkers, and therefore we have proposed a lower reference value (10th percentile) to use when applying RC adjustments.

The development of a new-regression adjustment approach is a major step forward because no universally accepted methods for accounting for inflammation when assessing micronutrient status currently exist to our knowledge. Although the WHO currently recommends the omission of individuals with elevated acute-phase protein concentrations (AGP or CRP) when calculating depleted iron stores using ferritin, this approach often results in a substantial portion of the data being lost from analyses in areas with high levels of inflammation. To our knowledge, there are also currently no WHO recommendations for adjusting other iron biomarkers (e.g., sTfR) or any vitamin A biomarkers (e.g., RBP or retinol). The lack of a consensus on how to adjust micronutrient biomarkers results in various approaches being used, which hinders the interpretability and comparability of micronutrient-deficiency data. It also continues to impede inflammatory biomarkers becoming a standard measurement in nutrition surveys.

The second major contribution of the BRINDA project is the characterization of potential factors associated with inflammation (5) and risk factors for anemia (10, 11) to better understand the drivers of these widespread phenomena. Inflammation is increasingly recognized as playing an important role in disease (55) and nutritional deficiencies, but little is known, to our knowledge, about the prevalence and demographic differences of inflammation in low- and middle-income countries. In contrast, anemia has long been recognized as a problem with recent global estimates in children (43%) and women (33%) revealing little to no reduction in anemia between 1995 and 2011 (56). Although poorly designed and implemented interventions have largely contributed to this lack of progress, an additional failure has been a poor understanding of anemia risk factors. Recently, a cause-specific attribution to anemia has been modeled with the use of estimation techniques that required a high degree of extrapolation (57). Although this is an important step forward, it underscores the need to assess risk factors directly. The body of work in the current supplement highlights the importance of the use of real data to assess risk factors for anemia and provides a conceptual framework for dealing with such analyses.

There are several strengths of the BRINDA project including its multipartner and collaborative nature, comparability of laboratory methods, and the large sample size from diverse countries. There are also important caveats about the data available. Data were aggregated from household-based surveys. The surveys were not designed a priori to answer the BRINDA project research questions, and as such, there were some differences in what variables were available and, in some cases, how variables were defined. In addition, although all WHO regions were represented in the BRINDA database, the data sets were selected with the use of a convenience sample and did not collectively represent the global population. Last, the findings cannot necessarily be applied to clinical aspects of inflammation and anemia in individual patients; thus, this investigation was focused at the population level (3).

Because the data sets were derived from cross-sectional surveys, we were unable to establish a temporal relation between the exposures and outcomes. In regard to the biomarker papers, this inability meant that a differentiation between true micronutrient deficiency and the body’s biological response to inflammation was not possible. In addition, we were unable to perform sensitivity analyses that compared adjustment approaches in the absence of gold-standard measurements of micronutrient biomarkers [iron (bone marrow aspirates) and vitamin A (liver reserves)]. However, as previously mentioned, the RC approach appears to better reflect the underlying linear relation between the micronutrient biomarker and inflammation.

Similar to the biomarker papers, the use of cross-sectional data was also a weakness for the inflammation and anemia papers because of the sequential nature of the relation between these outcomes and their risk factors. The BRINDA group was unable to fully capture the complex biological interplay between anemia, infections, inflammation, and micronutrient deficiencies. In addition, it was not possible to distinguish between different types of anemia in the present investigation. The measurement of the mean corpuscular volume, reticulocyte count, and haptoglobin may better elucidate the interrelations between the risk factors for anemia (58). There were also important risk factors for anemia and inflammation that were either not assessed or were not assessed in a sufficient number to be included in our models such as helminth infections, genetic disorders, and folate, vitamin B-12, vitamin D, and zinc deficiency (59–63).

The BRINDA project has made advances toward improving biomarker interpretation and identifying the major causes of anemia. It has also highlighted the value of the use of nutrition surveys to address important research questions at the same time as showing the need to conduct micronutrient surveys in a harmonized fashion and undertake longitudinal studies. Further research is needed to identify and compare different adjustment approaches, understand their biological implications, examine whether different types of infections modify the relation between inflammation and the micronutrient biomarkers, and explore adjustments to other micronutrient biomarkers. In addition, measuring more risk factors for anemia and performing structural equation modeling would lead to a better understanding of the factors that contribute to anemia, which, in turn, would result in more-effective programs.

In conclusion, this research has important policy and programmatic implications. Better prevalence estimates of iron deficiency and vitamin A deficiency would improve our ability to identify populations who are in greatest need of nutrition programs or populations who could potentially be at risk of excessive micronutrient intake. At the individual level, of particular importance is moving toward screening individuals for iron deficiency before supplementation. To mitigate the potential adverse effects of unnecessary iron supplementation, this screening requires the ability to assess iron status at the individual level, particularly in settings with high infection burden. Although it is unclear whether the results of BRINDA project can be applied to individuals, this project serves as a catalyst for future longitudinal and intervention studies on individual biomarker assessment (64). Above all, understanding the relative contribution of nutritional and other risk factors for anemia will improve the design of interventions that address the multifactorial causes of anemia. The insights that are gained from this project will help guide global and country investments to prevent and control micronutrient deficiencies and anemia globally.
